# The Effect of Intravenous Magnesium Sulfate and Lidocaine in Hemodynamic Responses to Endotracheal Intubation in Elective Coronary Artery Bypass Grafting: A Randomized Controlled Clinical Trial

**DOI:** 10.5812/aapm.15905

**Published:** 2014-06-21

**Authors:** Mehrdad Mesbah Kiaee, Saeid Safari, Gholam Reza Movaseghi, Mahmoud Reza Mohaghegh Dolatabadi, Masoud Ghorbanlo, Mehrnoosh Etemadi, Seyed Arash Amiri, Mohammad Mahdi Zamani

**Affiliations:** 1 Department of Anesthesiology and Pain Medicine, Iran University of Medical Sciences, Tehran, Iran; 2 Students' Scientific Research Centre, Tehran University of Medical Sciences, Tehran, Iran

**Keywords:** Coronary Artery Bypass Grafting, Endotracheal Intubation, Hemodynamic, Lidocaine, Magnesium Sulfate

## Abstract

**Background:**

There have been many concerns about alteration in hemodynamic parameters within and shortly after endotracheal intubation (ETI) in patients scheduled for elective coronary artery bypass grafting (CABG).

**Objectives:**

We compared the attenuation effect of magnesium sulfate and lidocaine on hemodynamic responses after ETI, in patients undergoing CABG.

**Patients and Methods:**

In this randomized controlled trial 150 patients undergoing elective CABG were enrolled. Included patients were randomly allocated to three groups and received lidocaine (1.5 mg/kg), magnesium sulfate (50 mg/kg within five minute), or normal saline, 90 seconds before ETI. Baseline hemodynamic parameters including heart rate (HR), systolic blood pressure (SBP), diastolic blood pressure (DBP), and mean arterial pressure (MAP) were recorded immediately before anesthesia induction, before intubation, immediately after intubation, and at second and fifth minutes after intubation.

**Results:**

The baseline hemodynamic variables had no deference among the three groups. HR between intubation and five minute after intubation was significantly lower in two groups received lidocaine or magnesium sulfate in comparison with placebo group. Lidocaine induced more than 20% decrease in HR and MAP immediately after intubation; hence, lidocaine group showed significant MAP reduction in comparison with the two other groups.

**Conclusions:**

Lidocaine induced hemodynamic instability but premedication of magnesium sulfate maintained hemodynamic stability after intubation. Therefore, in patients undergoing CABG who received high-dose intravenous analgesic for general anesthesia, the administration of magnesium sulfate might result in maintaining hemodynamic stability after ETI in comparison with lidocaine.

## 1. Background

The inappropriate response of hemodynamic parameters to endotracheal intubation (ETI) can increase perioperative and postoperative morbidity and mortality, particularly in patients with cardiovascular disease or those undergoing coronary artery interventions (1). A variety of medications have been suggested to control hemodynamic responses and prevent hemodynamic instabilities. In this regard, the beneficial effects of adding magnesium sulfate (MGS) to anesthetic drugs on improving intraoperative conditions such as minimizing hemodynamic instability were hypothesized (2). MGS inhibits catecholamines release from adrenal medulla and peripheral nerve endings and directly blocks catecholamines receptors. Therefore, MGS causes sympathetic block and indirectly causes dilated blood vessels and finally, reduces blood pressure (BP) (3). Studies on animals and human subjects have shown that the addition of intrathecal MGS may reduce the incidence of side effects observed after local anesthesia (4). Furthermore, intrathecal MGS injection would result in reducing postoperative pain and prolonged sensory blockade duration (5, 6). It has also been suggested that intravenous (IV) MGS can inhibit ETI-associated catecholamine release with that may result in stability of postoperative hemodynamic status (7). Moreover, MGS attenuates vasopressin-stimulated vasoconstriction and normalizes sensitivity to vasopressin (8). On the other hand, protective effects of IV lidocaine on intraoperative or postoperative hemodynamic changes are still unknown. Although some experimental studies could not show changes in hemodynamic condition following injection of lidocaine in anesthetized subjects (9), some others have emphasized the attenuation of cardiovascular responses to ETI by this drug (10).

## 2. Objectives

In the present study, we compared the attenuation effect of magnesium sulfate and lidocaine on hemodynamic responses after ETI in patients undergoing coronary artery interventions.

## 3. Patients and Methods

### 3.1. Study Design

In this randomized controlled double-blind study, we recruited 150 patients scheduled for elective coronary artery bypass grafting (CABG) under general anesthesia with ETI at Moheb Hospital, a private referral general hospital in Tehran, Iran. Those with American Society of Anesthesiologists (ASA) class more than II, obesity (body mass index > 30), difficulty in ETI, emergency surgery, left ventricular ejection fraction of less than 45% based on echocardiography reports, cardiac arrhythmias, renal dysfunction (serum creatinine level >2 mg/dL), history of hypersensitivity to lidocaine or MGS, and pregnant women were excluded. The study protocol was approved by the Ethics Committee of Moheb Hospital and informed consents were obtained from all patients. Patients were randomly allocated to three groups that received lidocaine (1.5 mg/kg), MGS (50 mg/kg within 5 minutes), or normal saline. Routine monitoring of electrocardiogram, pulse oximetry, non-invasive and invasive BP monitoring from left radial artery were commenced at operation room before surgery. For all the patients, IV line was secured with gauge-18 catheter and 3 to 4 mL/kg of isotonic crystalloid fluid was infused to check the IV line efficacy. Then the assigned drugs were administered intravenously 90 seconds before ETI. The syringes were filled with one of the study medications, namely, MGS, lidocaine, or normal saline (placebo), by an anesthesiology technician and handed over to the anesthesiologist who was blinded to the medications. Therefore, none of the patients and their anesthesiologists know the group that patients were allocated to.

### 3.2. Anesthesia Technique

Anesthetic management of patients in all study groups was similar. All patients were premedicated by intramuscular morphine (0.1 mg/kg) half an hour before surgery and ringer lactate infusion (8 to 10 mL/kg) was started. After preoxygenation with fraction of inspired oxygen (FIO_2_) of 1.0, anesthesia was induced with sufentanil (0.5-1 µg/kg), midazolam (20 µg/kg), etomidate (0.15-0.3 mg/kg), and cisatracurium (0.15-0.2 mg/kg). After three minutes, patients were intubated with the appropriate size, cuffed endotracheal tube (ETT). All intubations were performed by an experienced anesthesiologist. Anesthesia was maintained with infusion of midazolam (0.25-0.5 µg/kg/min) and fentanyl (0.03-0.1 µg/kg/min) and bolus administration of cisatracurium (0.03 mg/kg) every 30 minutes required. In all study groups, ventilation was stopped during the period of cardiopulmonary bypass. At the end of bypass, lungs were manually reinflated under direct observation using a continuous positive airway pressure CPAP of 20 cm H_2_O.

### 3.3. Study Measurements

Hemodynamic parameters of patients including systolic BP (SBP), diastolic BP (DBP), mean arterial pressure (MAP), and heart rate (HR) were recorded immediately before anesthesia induction, before ETI, immediately after ETI, and at second and fifth minutes after ETI.

### 3.4. Study Definitions

Hemodynamic stability was determined as changes between -20% to +20% from baseline BP. Diagnosis of hypertension was determined by positive history of antihypertensive drugs use. Positive history of cigarette smoking was defined as smoking cigarette for more than ten pack-years.

### 3.5. Statistical Analysis

According to the similar previous study on 60 patients (30 in each group), we determined that a sample size of about 50 in each group would be sufficient to detect the difference in hemodynamic indices by considering a standard deviation of 20% of mean difference as the minimum detectable difference of means hemodynamic indices among three groups, a power of 95%, and a significance level of 5%.

All statistical analyses were performed using SPSS software (version 20.0, SPSS Inc., Chicago, IL, the USA). Data were expressed as mean ± standard deviation for quantitative variables and were summarized by absolute frequencies and percentages for categorical variables. Categorical variables were compared using Chisquare test or Fisher’s exact test. Quantitative variables were also compared with one-way ANOVA test. Differences in the trend of changes in hemodynamic parameters among study groups were examined using the repeated measure ANOVA test. Statistical significance was determined as a P value < 0.05.

## 4. Results

Demographic data were similar among three groups (Table 1). The baseline hemodynamic parameters were not different among three groups (P > 0.05). Table 2 represents the hemodynamic parameters of three groups at the study time points. HR was significantly lower from ETI time through the fifth minute after ETI in those receiving lidocaine or MGS in comparison with the placebo group. There were no trends in the hemodynamic parameters in comparison to the baseline values in MGS group; all parameters were reduced from anesthesia induction through ETI time, then elevated after ETI by about 10% from baseline, and then gradually decreased until the fifth minutes after ETI (Table 2). The trends of changes in the parameters between placebo and MGS groups were not significantly different. Inter-group comparison showed significantly lower DBP and MAP immediately after ETI in the lidocaine group in comparison with two other groups. 

**Table 1.** Demographic Data and Clinical Data of the Study Participants ^a,b^

Characteristics	Control Group (No = 52)	Lidocaine Group (No = 48)	MgSO4 Group (No = 50)	P value
**Sex, Male/Female**	32/20	32/16	34/16	0.769
**Age, y**	59.2 ± 12.2	60.1 ± 5.4	58.6 ± 8.6	0.770
**BMI, kg/m** ^**2**^	26.4 ± 4.3	25.7 ± 4.7	24.8 ± 4.0	0.199
**Ejection Fraction,%**	48.0 ± 4.6	49.3 ±9.0	49.5 ± 5.7	0.462
**Valvular Heart Disease **	0 (0.0)	3 (6.3)	2 (4.0)	0.209
**Myocardial Infarction **	12 (23.1)	11 (22.9)	10 (20.0)	0.916
**Hypertension **	32 (61.5)	30 (62.5)	27 (54.0)	0.640
**Diabetes Mellitus**	16 (30.8)	20 (42.6)	18 (36.0)	0.476
**Smoking **	10 (19.2)	8 (16.7)	12 (24.0)	0.653
**Medication**				
**Beta-Blocker**	51 (98.1)	44 (91.7)	44 (88.0)	0.141
**Calcium-Blocker**	18 (34.6)	17 (35.4)	17 (34.0)	0.989
**ACE-I**	35 (67.3)	30 (62.5)	36 (72.0)	0.605
**Nitrates**	42 (80.8)	38 (79.2)	40 (80.0)	0.980

^a^ Abbreviations: BMI, body mass index; and ACE-I, angiotensin converting enzyme-inhibitor.

^b^ Data are presented as mean ± SD or No. (%).

**Table 2.** Hemodynamic Status During Study^a^

Characteristic	Placebo Group (No = 52)	Lidocaine group (No = 48)	MgSO4 Group (No = 50)	P value
**Systolic Blood Pressure**, **mmHg**				
Before Induction	152.6 ± 23.7	160.2 ± 24.8	154.7 ± 22.8	0.266
Before Intubation	100.5 ± 20.4	97.7 ± 16.9	115.5 ± 25.3	0.437
After Intubation	130.7 ± 34.0	119.9 ± 30.2	120.6 ± 34.5	0.095
2 min After Intubation	146.5 ± 13.0	129.2 ± 28.9	126.4 ± 26.8	0.398
5 min After Intubation	113.3 ± 18.4	111.4 ± 24.0	112.5 ± 19.5	0.896
**Diastolic Blood Pressure, mmHg**				
Before Induction	78.7 ± 12.4	77.1 ± 10.6	77.5 ± 10.5	0.756
Before Intubation	55.6 ± 10.0	53.0 ± 8.8	66.2 ± 9.2	0.161
After Intubation	74.3 ± 17.4	66.7 ± 14.2	70.9 ± 18.9	0.018
2 min After Intubation	67.8 ± 12.0	64.2 ± 12.6	66.0 ± 13.9	0.388
5 min After Intubation	64.0 ± 9.4	61.0 ± 10.6	64.2 ± 10.6	0.148
**Mean Arterial Pressure, mmHg**				
Before Induction	104.5 ± 18.0	106.2 ± 16.2	105.2 ± 14.8	0.870
Before Intubation	70.1 ± 13.2	68.6 ± 12.3	77.7 ± 11.6	0.606
After Intubation	92.4 ± 23.9	82.4 ± 20.9	84.4 ± 24.4	0.049
2 min After Intubation	86.2 ± 17.7	81.4 ± 17.1	81.2 ± 19.1	0.238
5 min After Intubation	79.9 ± 12.3	77.7 ±13.8	78.4 ± 14.3	0.702
**Heart Rate, beat/min**				
Before Induction	82.6 ± 17.7	79.9 ± 14.7	70.5 ± 15.5	0.413
Before Intubation	69.0 ± 14.0	66.2 ± 12.0	65.7 ± 13.0	0.384
After Intubation	80.9 ± 16.0	74.3 ± 14.3	75.3 ± 15.2	0.048
2 min After Intubation	77.5 ± 14.7	71.7 ± 14.5	71.1 ± 13.4	0.046
5 min After Intubation	73.8 ± 13.8	68.3 ± 13.2	67.6 ± 11.8	0.034

^a^ Data are presented as mean ± SD

## 5. Discussion

The present study revealed that in patient undergoing CABG who had received high-dose IV analgesic for general anesthesia, the administration of MGS resulted in maintaining hemodynamic stability after ETI. These effects were significant in comparison with lidocaine, which induced more than 20% reduction in SBP, DBP, and MAP, and placebo, which caused more increase in SBP, DBP, and MAP; however, both Lidocaine and MGS may lead to similar reduction in HR after ETI. The mechanism of the action of both drugs is obviously multifactorial. The different possible mechanisms of action of MGS have been discussed. It was reported that MGS can induce endothelium-derived nitric oxide production that mediates the relaxation of vascular smooth muscles through its vasodilatory effect (11). In addition, MGS acts as a vasodilator by increasing the synthesis of prostacyclin as well as inhibiting angiotensin converting enzyme activity (12). The mechanism of action is unclear, but its blocking effects on calcium channels and N-methyl-D-aspartate (NMDA) receptors seems to play an important role (12, 13). 

In this study, the MGS group received 50-mg/kg MGS as an IV bolus in a five-minute period before the anesthesia induction. This regimen resulted in a steady and smooth reduction in MAP and reduced HR with no episodes of severe hypotension, which is similar to previous studies (14, 15). MGS was chosen since it is a vasodilator with minimal myocardial depression (16) which is the dose-dependent depressant effect on cardiac contractility. It has been shown that the depressant effect of MGS on cardiac function is offset by lowering the systemic vascular resistance (SVR) and hence, MGS maintains cardiac pump function (17). We know that MAP is determined by cardiac output (CO), SVR, and central venous pressure (CVP) according to the following equation, which is based on the association among flow, pressure, and resistance: MAP = (CO × SVR) + CVP; CVP is usually at or near 0 mmHg; therefore, this formula is often simplified to: MAP ~ CO × SVR. Hence, changes in either CO or SVR will affect MAP. In Khajavi et al. study on 32 major non-laparoscopic gastrointestinal surgeries, premedication with 40 mg/kg bolus and 10 mg/kg intraoperative infusion of MGS decreased both intraoperative CO and SVR in comparison with placebo and thus, MAP decreased during operation in MGS group while increased in placebo group (P < 0.001) (18). Shin et al. investigated the lower bolus dose of MGS (10 and 20 mg/kg) prior to muscle relaxant and detected the attenuating effect of MGS on rocuronium injection-associated pain as well as laryngoscopy and ETI-associated cardiovascular changes (19). In addition, the role of preoperative MGS administration in controlling intraoperative hypertension and reducing the intraoperative variability of arterial pressure has been studied in patients with hypertension undergoing cataract surgery with local anesthesia (20). It has been shown that MGS, as a safe drug without any hemodynamic instability, is as effective as nicardipine in controlling arterial pressure during cardiac procedures (21) and shortens postoperative time for extubation in elective CABG surgeries (22). Some previous studies have demonstrated that the infusion of 1.5 to 2 mg/kg of lidocaine from the fifth to the second minute before laryngoscopy can blunt the increase in HR, SBP, MAP and catecholamine levels associated with intubation (23-25). Another studies found that IV lidocaine with similar dosages failed to control the hemodynamic response following laryngoscopy and ETI (26). This controversy may be referred to the importance of timing of the lidocaine administration. Considering the mechanism of lidocaine, inhibiting the sympathetic response associated with tracheal stimulation appears to result from an increased threshold for airway stimulation, central inhibition of sympathetic transmission, and direct depression of cardiovascular responses (27).

In a recent study by Panda et al., patients with hypertension undergoing elective surgery under general anesthesia were studied. A total of 80 patients were randomly allocated to three groups of MGS infusion at dose of 30, 40, or 50 mg/kg before induction of anesthesia, and a group of 1.5 -mg/kg lidocaine bolus 90 seconds before intubation. MAP was maintained within normal limits with 30 -mg/kg MGS while 40 and 50 mg/kg of MGS induced a significant decrease in MAP. A total of six patients with 40 mg/kg and ten patients with 50 mg/kg of MGS required interventions. Only one patient with lidocaine required intervention. On the other hand, anyone with 30-mg/kg MGS required intervention. Panda et al. concluded that 30-mg/kg MGS was better than lidocaine administration in patients with hypertension and regarding dose of MGS, a further step-up in the dose of MGS from 30 to 50 mg/kg might cause significant hypotension and more medical expenses (28). According to our findings, administration of MGS helps to maintain BP at the lower limit of normal without adverse effect on BP or HR. Regarding the effects of lidocaine on BP responses, our study showed that the administration of lidocaine (1.5 mg/kg) before intubation resulted in decreased BP and HR in comparison with placebo. Nooraei et al. showed similar results but they concluded that MGS might increase the HR (29). Although both MGS and lidocaine might reduce HR, MGS is preferred because of its beneficial effects on maintaining BP after intubation in CABG; moreover, MGS was safer than lidocaine in maintaining BP stability after ETI.

Patients suffering from cardiovascular disease, especially those who are candidate for CABG, need hemodynamic stability and MGS stabilizes their BP more effectively than lidocaine. For such patients, anesthesia induction with high-dose IV analgesic would lead to BP suppression while MGS attenuate this effect. Lidocaine induces more than 20% suppression from baseline after one minute of ETI while MGS provides hemodynamic stability during the five minutes after ETI.
